# Probing the applicability of autotransporter based surface display with the EstA autotransporter of *Pseudomonas stutzeri* A15

**DOI:** 10.1186/1475-2859-11-158

**Published:** 2012-12-13

**Authors:** Toon Nicolay, Lynn Lemoine, Elke Lievens, Sam Balzarini, Jos Vanderleyden, Stijn Spaepen

**Affiliations:** 1 Centre of Microbial and Plant Genetics, KU Leuven, Kasteelpark Arenberg 20, 3001, Heverlee, Belgium

**Keywords:** Autodisplay, Proteinase K, Secretion, Type V secretion system, Outer membrane protein, Beta barrel

## Abstract

**Background:**

Autotransporters represent a widespread family of secreted proteins in Gram-negative bacteria. Their seemingly easy secretion mechanism and modular structure make them interesting candidates for cell surface display of heterologous proteins. The most widely applied host organism for this purpose is *Escherichia coli*. *Pseudomonas stutzeri* A15 is an interesting candidate host for environmentally relevant biotechnological applications. With the recently characterized *P. stutzeri* A15 EstA autotransporter at hand, all tools for developing a surface display system for environmental use are available. More general, this system could serve as a case-study to test the broad applicability of autotransporter based surface display.

**Results:**

Based on the *P. stutzeri* A15 EstA autotransporter β-domain, a surface display expression module was constructed for use in *P. stutzeri* A15. Proof of concept of this module was presented by successful surface display of the original EstA passenger domain, which retained its full esterase activity. Almost all of the tested heterologous passenger domains however were not exposed at the cell surface of *P. stutzeri* A15, as assessed by whole cell proteinase K treatment. Only for a beta-lactamase protein, cell surface display in *P. stutzeri* A15 was comparable to presentation of the original EstA passenger domain. Development of expression modules based on the full-length EstA autotransporter did not resolve these problems.

**Conclusions:**

Since only one of the tested heterologous passenger proteins could be displayed at the cell surface of *P. stutzeri* A15 to a notable extent, our results indicate that the EstA autotransporter cannot be regarded as a broad spectrum cell surface display system in *P. stutzeri* A15.

## Background

To cross the subsequent hurdles posed by the inner membrane, the periplasm and the outer membrane, Gram-negative bacteria have evolved at least seven different protein secretion mechanisms [1,2]. One widespread family of secreted proteins is constituted by autotransporters (ATs). These ATs represent a subset of the type V secretion system. This system is often regarded as the most simple secretion apparatus of Gram-negative bacteria. ATs possess a modular structure consisting of an N-terminal signal peptide for targeting to the Sec translocon and subsequent translocation across the inner membrane, a passenger domain which represents the (often secreted) extracellular part and a C-terminal β-barrel domain which anchors the protein in the outer membrane. The passenger domain and the β-barrel domain are separated by an α-helical linker domain [3]. The term AT was based on the initial view that no accessory proteins are required for transport of the passenger domain to the cell surface (and subsequent secretion). Yet, evidence shows that at least the Bam complex also fulfills an important role in this process [4].

Research in ATs originates from the role of most characterized ATs as virulence factors [5]. Apart from this, ATs are mainly of interest because replacement of the passenger domain with a heterologous protein makes them suitable candidates for cell surface display, a process termed autodisplay. Amongst others, it has been applied in vaccine development, library screening and whole-cell biocatalysis [6]. The most recent use is the extracellular accumulation of recombinant proteins [7,8]. Often this system is regarded as a universal and broadly applicable cell surface display system posing minimal requirements to the passenger domain [6,8].

The biotechnological use of AT proteins has been mainly focused on *Escherichia coli* as host organism. For environmentally relevant biotechnological applications however, a more robust host is needed. The use of a different host organism for such applications is nevertheless limited to a few reports addressing metal remediation with *Cupriavidus metallidurans* CH34 [9,10] or *Pseudomonas putida*[11] for which the researchers applied the heterologous AT IgA of *Neisseria gonorrhoeae*. The use of an autologous AT, instead of a heterologous AT, might however be more appropriate. At least for *E. coli*, it has been suggested that the actual breakthrough for autodisplay has been initiated by the use of a for *E. coli* autologous AT [12].

Because of their involvement in environment-related processes, *P. stutzeri* strains represent good candidates for use in environmental applications [13]. We recently characterized the *P. stutzeri* A15 EstA AT [14], a close homologue of EstA of *Pseudomonas aeruginosa* PAO1 for which the full-length crystal structure is known [15]. They both are members of the GDSL ATs which represent a distinct family among the AT family. Features that distinguish their passenger domains from the majority of the ATs are their lipolytic activity, the permanent covalent attachment to the β-domain after surface display and the absence of the right-handed parallel β-helix structure [16]. An expression module based on the *Pseudomonas putida* EstA β-barrel domain was used to display a β-lactamase [17] and *Pseudomonas* and *Burkholderia* lipases at the cell surface of *E. coli*[18]. Becker *et al.* developed an expression module based on the entire EstA AT of *P. aeruginosa* for the cell surface display of lipolytic enzymes [19]. With the same expression module also a foldase was displayed at the cell surface [20].

We aimed to investigate the broad applicability of cell surface display of heterologous passenger domains using the AT system. As an interesting case-study we developed a *P. stutzeri* A15 EstA AT based expression module for the surface display of environmentally relevant proteins in *P. stutzeri* A15. This bacterium was isolated from the roots of paddy rice and later on classified as *P. stutzeri*[21,22]*.* The design of our β-domain based expression module could successfully be tested by the surface display of the original EstA passenger domain. This proof of concept contrasted with the encountered difficulties when heterologous passenger domains were used. We could show that the majority of these proteins were not displayed at the cell surface of *P. stutzeri* A15. The construction of two different expression modules based on the full-length EstA AT did not substantially solve these problems. Consequently, we conclude that the EstA AT cannot be regarded as a broad applicable cell surface display system in *P. stutzeri* A15.

## Results

### An EstA β-domain based expression module and the original EstA passenger domain as proof of concept

**Figure 1:**
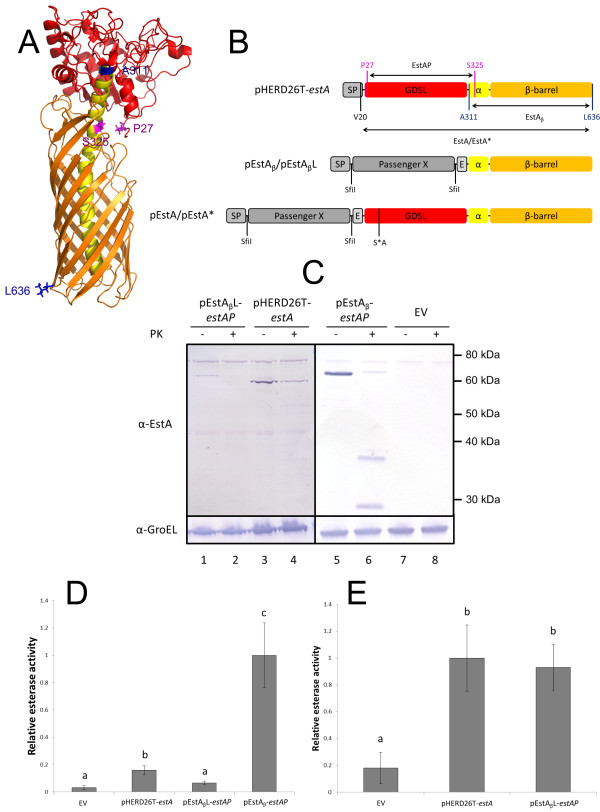
**Surface display of the EstA passenger domain. **(**A**) Structural model of *P. stutzeri *A15 EstA (from D24 to L636) constructed using I-TASSER [23]. (**B**) Schematic representation of proteins expressed from pHERD26T-*estA *(EstA), pEstA_β_/pEstA_β_L and pEstA/pEstA*. (**AB**) Residues delineating the part of EstA used for constructing pEstA_β_/pEstA_β_L (EstA_β_) are in blue (A311 and L636). Residues marking the esterase passenger domain (EstAP) are coloured in magenta (P27 and S325). Residues for generating the full-length AT based constructs pEstA/pEstA* (V20, black and L636, blue; see further) have been depicted. The difference between pEstA and pEstA*, mutagenesis of the catalytic serine residue (S*A), has been indicated. The GDSL esterase passenger domain (GDSL), the α-helical linker domain (α) and the β-barrel domain have been depicted in red, yellow and orange respectively, signal peptide (SP), E-epitope tag (E), SfiI restriction site (SfiI). (**C**) Induced cells of *P. stutzeri *A15 pHERD26T-*estA*, pEstA_β_*estAP*, pEstA_β_L-*estAP *or empty vector (EV) were treated with proteinase K (PK +) or mock-treated (PK -) and analyzed with Western blot using anti-EstA serum (α-EstA) or anti-GroEL (α-GroEL). (D-E) Relative esterase activity of (**D**) cells of *P. stutzeri *A15 pHERD26T-*estA*, pEstAβ-*estAP*, pEstAβL- *estAP *or empty vector (EV) or (**E**) membrane fractions containing equal amounts of EstA/EstAP (Additional file 1: Figure S1). Data represent the mean of three independent repeats ± 95% confidence interval. The significance level (P < 0.05) as determined by one-way ANOVA with Student-Newman-Keuls post-hoc analysis, is indicated with a letter code. Molecular weight markers (kDa) are indicated at the side.

The recently published crystal structure of the EstA homologue of *P. aeruginosa*[15] served as a template to construct a structural model using the I-TASSER server [23] depicted in Figure 1A. The model allowed for the structurally informed design of two *P. stutzeri* A15 EstA based expression modules. Although still under debate [24,25], recent research suggests that only the β-barrel and the α-helical linker are sufficient for AT based surface display [8,26]. Therefore the designed expression modules, integrated in the pHERD26T backbone, contain a transport unit consisting of the β-barrel and the α-helical linker (from residue A311 to L636, residue numbering starting at the GTG start codon; EstA_β_ on Figure 1B) of *P. stutzeri* A15 EstA as derived from the structural model. These expression modules also contain the *P. stutzeri* A15 EstA signal peptide. Furthermore two SfiI sites which allow directional cloning of different passenger domains and an E-epitope tag for detection were cloned between the signal peptide and the transport unit (Figure 1B). The signal peptide cleavage site was not affected compared to the wild type EstA when assessed by SignalP [27]. Both the SfiI sites and the E-epitope tag have previously been used to construct a full length *P. aeruginosa* system for the surface display of lipases [19]. The two expression modules were named pEstA_β_ and pEstA_β_L, and express identical ORFs but differ at the 5^′^ untranslated region (UTR) preceding the ORF. The 5^′^ UTR of pEstA_β_L is identical to the 5^′^ UTR of pHERD26T-*estA*, which expresses the EstA AT, and was therefore expected to have similar expression levels as the latter. As a preliminary test, the *P. stutzeri* A15 EstAP passenger domain (from residue P27 to S325, EstAP on Figure 1B) was cloned as the passenger domain in the expression modules yielding pEstA_β_*estAP* and pEstA_β_L-*estAP*. The EstAP fusion protein expressed from these plasmids only differs from EstA, which was expressed from pHERD26T-*estA*, by the presence of the E-epitope tag and the SfiI sites. A schematic representation of EstA derived from pHERD26T-*estA* and a fusion protein expressed from the expression modules pEstA_β_ and pEstA_β_L is depicted in Figure 1B.

The EstAP fusion proteins derived from pEstA_β_-*estAP* and pEstA_β_L-*estAP* as well as EstA from pHERD26T-*estA* were produced in *P. stutzeri* A15 cells. Both expression and cell surface display could be confirmed for all proteins by analyzing samples derived from whole cell proteinase K assays with SDS-PAGE and Western blot using α-EstA serum (Figure 1C). Cell integrity in these samples was monitored by testing GroEL as a negative control protein. Furthermore, the functionality of the fusion proteins was tested by assessing the esterase activity of whole cells (Figure 1D). The data revealed the expected lower esterase activity of cells containing pHERD26T-*estA* or pEstA_β_L-*estAP* as compared to cells containing pEstA_β_-*estAP*. This was expected since the 5^′^ UTR of these constructs is different. More surprising was the observation that the whole cell esterase activity differed between cells containing pHERD26T-*estA* and pEstA_β_L-*estAP*. The esterase activity level of the latter did not even significantly differ from the empty vector (EV) negative control. Western blot analysis however revealed that the protein level of EstAP derived from pEstA_β_L-*estAP* is much lower than the level of EstA (Figure 1C). Moreover, the analysis of the esterase activity of membrane fractions (MFs) containing equal amounts of EstA or EstAP (Additional file 1: Figure S1) revealed that both proteins showed similar esterase activities (Figure 1E) and that this activity was membrane-associated.

Despite the similarities between the EstAP fusion proteins and EstA, we could observe a difference in stability of the passenger domain. We have previously shown that also the passenger domain of EstA and not only the β-barrel domain shows a heat modifiability shift, indicative of its relatively high stability [14]. Heat modifiability analysis showed that this shift could not be seen for the passenger domain of the EstAP fusion proteins. This is indicative of a less stable passenger domain in the EstAP fusion proteins (Additional file 2: Figure S2). The presence of the E-tag and the SfiI sites are likely to be responsible for the lowered stability of the passenger domain of EstAP compared to the one of EstA. Presumably they are also accountable for the difference in protein levels between pHERD26T-*estA* and pEstA_β_L containing cells.

Altogether these results indicate that the insertion of additional elements for the construction of an EstA based expression module do not abolish the functional surface display of the autologous passenger domain in *P. stutzeri* A15. The low protein levels obtained with pEstA_β_L favor the use of pEstA_β_ in further experiments.

### Limitations for cell surface display of heterologous passenger proteins

Next, we intended to verify the universality of the pEstA_β_ expression module. Hereto, the coding sequences of several different passenger domains were cloned into the SfiI sites of pEstA_β_ (Additional file 3: Table S1). Because of a general interest in addressing quorum sensing dependent biofilm formation, we assessed the cell surface display of three different lactonases: a *Bacillus* AiiA homologue named AiiA_soil_ and the related lactonases of *Agrobacterium tumefaciens* AiiB and AttM. To verify the more general applicability of the expression module also other proteins were examined: the fluorescent proteins mCherry, eGFP and yEVenus and the periplasmic protein Bla which has beta-lactamase activity.

**Figure 2:**
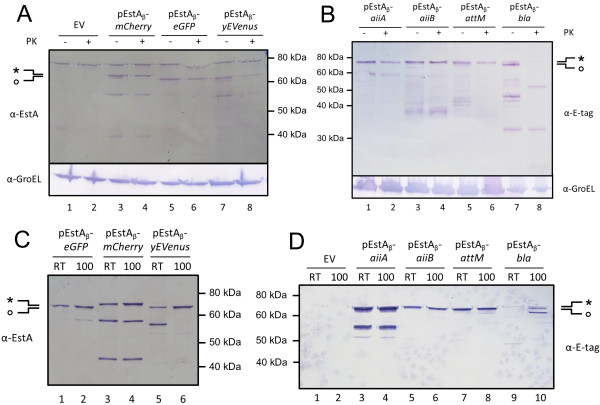
**EstA β-domain mediated surface display of heterologous passenger domains. **(**A**-**B**) Induced cells of *P. stutzeri *A15 pEstA_β_-*mCherry*/*eGFP*/*yEVenus*/*aiiA*/*aiiB*/*attM*/*bla* or empty vector (EV) were treated with proteinase K (PK +) or mock-treated (PK -). Samples were analyzed with Western blot using anti-EstA serum (α-EstA), anti-E-tag antibodies (α-E-tag) or anti-GroEL antibodies (α-GroEL). (**C-D**) Heat modifiability analysis of proteins in the membrane fractions of samples in (**A**-**B**). SDS-PAGE samples were incubated at room temperature (RT) or 100°C and analyzed with Western blot using anti-EstA serum (α-EstA). The band with the higher apparent molecular weight, containing the signal peptide, is indicated with an ‘*’. The band with the lower apparent molecular weight, without the signal peptide is indicated with an ‘o’. Molecular weight markers are indicated at the side of the panels.

Fusion protein production and cell surface display in *P. stutzeri* A15 was examined using whole cell proteinase K assays and subsequent analysis of the samples with SDS-PAGE and Western blot using α-E-tag antibodies or α-EstA serum (Figure 2A, B and Additional file 4: Figure S3). In most cases two different bands could be identified with a small difference in apparent molecular weight. The band with the higher apparent molecular weight (indicated with a ‘*’ in Figure 2), which represented the majority of the fusion proteins, was inaccessible for proteinase K. The band with the lower apparent molecular weight (indicated with an ‘o’ in Figure 2), which is much less abundant than the band with the higher molecular weight, was accessible for proteinase K. Membrane fractions were isolated to assess the heat modifiability of these different bands (Figure 2C and D). The results showed that the band with the higher apparent molecular weight was not heat modifiable. In some cases the lower band did show a heat modifiability, in other cases this band is too faint to clearly visualize a potential heat modifiability shift. In combination with the evidence obtained from the whole cell proteinase K treatment assays this implies that the band with the lower apparent molecular weight was correctly folded in the MF and displayed at the cell surface. More importantly, the majority of the fusion proteins was not present in a correctly folded manner in the MF and was also not displayed at the cell surface. One notable exception was pEstA_β_-*bla* in which case the majority of the fusion protein was present as the band with the lower apparent molecular weight that was proteinase K accessible (Figure 2B, lanes 7 and 8) and heat modifiable (Figure 2D, lanes 9 and 10).

The most obvious explanation for the difference in molecular weight between the two protein bands is the presence of the signal peptide in the protein band with the highest molecular weight. Therefore, the presence of this protein band in the MF indicates that these proteins are stuck at the inner membrane or represent protein aggregates that are too small to be precipitated by the initial clearance step of the cell fractionation protocol. Both explanations are in accordance with the absence of a heat modifiability shift of the proteins and their inaccessibility for proteinase K. In some cases also smaller sized specific protein bands could be identified, indicating either way translation intermediates or breakdown products.

Alltogether the results obtained for the heterologous passenger domains strongly differ from the results for the successfully displayed original EstA passenger domain. The results provide evidence that in almost all cases only a limited amount of the in *P. stutzeri* A15 expressed fusion proteins with a heterologous passenger domain can be displayed at the cell surface.

### Presence of the original AT passenger domain does not substantially improve surface display of heterologous passenger domains

Because of the problems associated with the pEstA_β_ expression module we decided to construct a full-length AT based expression module. Moreover, recently published work suggested a promoting role for the autologous passenger domain in AT based surface display of heterologous passenger domains [25]. Also, previous work on surface display based on EstA of *P. aeruginosa* made use of a full-length AT based expression module [19,20].

The pEstA and pEstA* expression modules consist of the full length EstA of *P. stutzeri* A15 (from residue V20 to residue L636, see Figure 1B). In the pEstA* plasmid, the catalytic serine of EstA was substituted for an alanine by site directed mutagenesis. Besides this mutation, both plasmids are identical to the pEstA_β_ plasmid containing the *P. stutzeri* A15 EstA signal peptide, two SfiI sites and an E-epitope tag. The same passenger domains as used for the pEstA_β_ experiments were cloned in pEstA and pEstA* (Additional file 3: Table S1). A schematic representation of a fusion protein expressed from these plasmids has been depicted in Figure 1B.

**Figure 3:**
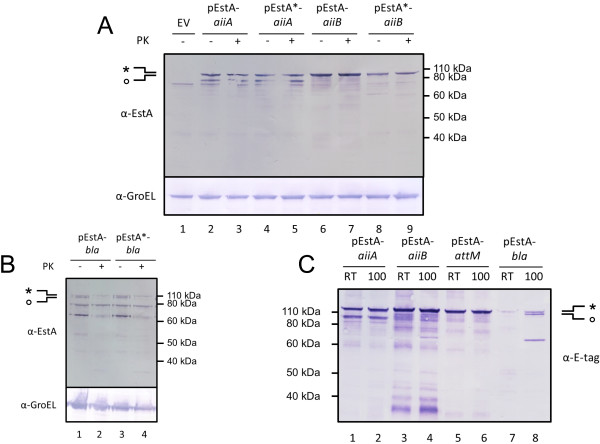
**EstA β-domain mediated surface display of heterologous passenger domains. **(**A**-**B**) Induced cells of *P. stutzeri *A15 pEstA/pEstA*-*aiiA*/*aiiB*/*bla *or empty vector (EV) were treated with proteinase K (PK +) or mock-treated (PK -). Samples were analyzed with Western blot using anti-EstA serum (α-EstA) or anti-GroEL antibodies (α-GroEL). Similar Western blots for *P. stutzeri* A15 pEstA/pEstA*-*aiiA*/*aiiB*/*attM/mCherry/eGFP/yEVenus/bla* using anti E-tag antibodies are given in Additional file 6: Figures S5-S8. (**C**) Heat modifiability analysis of proteins in the membrane fractions of pEstA samples in (**A**-**B**). SDS-PAGE samples were incubated at room temperature (RT) or 100°C and analyzed with Western blot using anti E-tag antibodies (α-E-tag). Western blots for pEstA-*mCherry/eGFP/yEVenus* and pEstA* samples are given in Additional file 7: Figure S9-S11. The band with the higher apparent molecular weight, containing the signal peptide, is indicated with an ‘*’. The band with the lower apparent molecular weight, without the signal peptide is indicated with an ‘o’. Molecular weight markers are indicated at the right side of the panels.

The production of the fusion proteins and their cell surface display in *P. stutzeri* A15 was assessed using whole cell proteinase K assays and analysis of the samples with SDS-PAGE and Western blot (Figures 3A-B and Additional files 5: Figures S4-S7). In general, the results are similar to the ones obtained for the pEstA_β_ derived fusion proteins. Again, the bulk of the fusion proteins was inaccessible for proteinase K. When analyzing the MF, the inaccessible proteins also did not show a heat modifiability shift while the less abundant proteinase K accessible proteins did show a heat modifiability shift (Figures 3C and Additional files 6: Figures S8-S10). Also here, the exception was pEstA-*bla*/pEstA*-*bla* of which the greater part of the fusion proteins was properly folded in the MF (Figure 3C and Additional file 6: Figure S8) and displayed at the cell surface (Figure 3B and Additional file 5: Figure S7). In general, lower bands that did not show heat modifiability presumably represent translation intermediates or breakdown products.

These data suggest that the problems associated with the EstA AT based surface display using the β-barrel domain in *P. stutzeri* A15 cannot substantially be alleviated by including the original passenger domain in the expression module.

### The beta-lactamase passenger domain is present in an enzymatically active conformation

**Figure 4:**
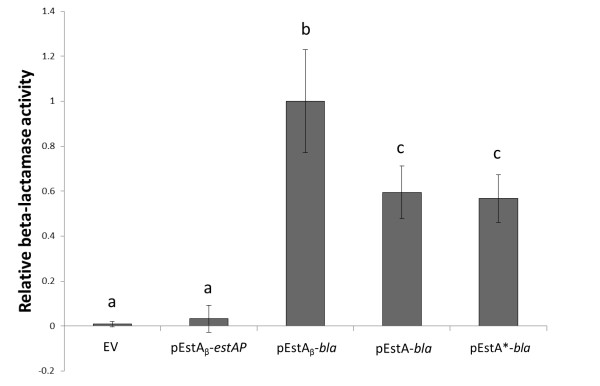
**β-lactamase activity of *P. stutzeri *A15 pEstA**_**β**_-*bla*, pEstA-*bla *and pEstA*-*bla. *Relative β-lactamase activity of whole cells of *P. stutzeri* A15 pEstA_β_-*estAP*, pEstA_β_-*bla*, pEstA-*bla*, pEstA*-*bla* or empty vector (EV) assessed with nitrocefin as the substrate. Data represent the mean of three independent repeats ± 95% confidence interval. The significance level (P < 0.05) as determined by one-way ANOVA with Student-Newman-Keuls as post-hoc analysis is indicated with a letter code.

Surface display does not imply that the passenger domain is also in an active conformation. As described above (Figure 1D), the EstA passenger domain retained its esterase activity when displayed at the cell surface with the pEstA_β_ expression module. The whole cell proteinase K and heat modifiability assays showed that only the beta-lactamase passenger domain was substantially displayed at the cell surface of *P. stutzeri* A15. Therefore the beta-lactamase activity of whole cells containing the pEstA_β_-*bla*, pEstA-*bla* and pEstA*-*bla* plasmids was analyzed using nitrocefin as a substrate. This revealed that in all three cases a significant beta-lactamase activity could be detected as compared to the EV negative control and pEstA_β_-*estAP* (Figure 4).

## Discussion

Up to now, various ATs have been used for the cell surface display of in total over thirty passenger domains [12]. In the past, the versatility and universality of AT based cell surface display has been emphasized [6,28]. Even more recently it was pointed out that this technology could be used for the extracellular accumulation of proteins with a diversity in size, structure and function [8]. With this in mind, we set out to test the broad applicability of AT based cell surface display using the recently characterized autologous EstA AT [14] in *P. stutzeri* A15 as a test-case.

In the first experiments, an expression module based on the EstA β-barrel and α-helix was constructed. Whole cell proteinase K assays and esterase activity assays validated the expression module by showing the successful cell surface display of the original EstA passenger domain. Also cell surface display of a functional beta-lactamase as the passenger domain could be attained. So far, our results are in line with previous papers describing AT based cell surface display of esterases/lipases [18,19,29,30] and a beta-lactamase [17] in *E. coli*. However, the same expression module failed to successfully display the majority of the fusion proteins with one of three different lactonases, AiiA_soil_, AiiB or AttM, as passenger domain. Also when one of the fluorescent proteins mCherry, eGFP or yEVenus was used as passenger domain, no successful cell surface display could be realized. Most reports focusing on passenger domain structure have looked at formation of disulfide bonds within the passenger domain in the periplasm and the compatibility with surface display. Recent research seems to have reached a consensus on this point suggesting that only disulfide bonds between closely spaced cysteine residues in the passenger domain can be tolerated to achieve successful surface display [31,32]. The crystal structures of the fluorescent passenger domains reveal a β-barrel fold without disulfide bonds [33-35]. The same is true for the crystal structure of the AiiA lactonase of *Bacillus thuringiensis*[36] and on the basis of homology also for the lactonases used in this study. The only passenger domain that could successfully be surface displayed was a TEM β-lactamase. Interestingly, the crystal structure of the TEM-1 β-lactamase [37], contains one disulfide bond. The overall structure of this protein reveals a more globular structure which resembles most the structure of the original GDSL passenger domain. However, the globular structure is most likely not the main reason for surface display since β-lactamases have already been used as passenger domain for surface display using ATs with a completely different passenger domain structure [25,38]. Whereas the surface display of β-lactamases is in line with our findings, the display of the mCherry protein at the cell surface using the Pet AT [8] and the mRFP1 protein using the YfaL AT [7] is in contrast with our findings. In both studies [7,8] cell surface display was achieved in *E. coli*.

A minimal translocation unit for AT based cell surface display, consisting of the β-barrel domain and the α-helical linker domain, has been described for several ATs [39-43] including the EstA AT of *P. putida*[17]. More recently however, this original view was debated by the finding that the cell surface expression of heterologous proteins was increased when the full-length IcsA AT was used rather than only the β-domain [25]. This was supported by work on the Hbp AT where an intact β-stem was needed for efficient extracellular expression of the ESAT6 antigen [24].

Because of these reports and other publications in which a full-length EstA AT of *P. aeruginosa* was applied [19,20] we designed full length EstA expression modules based on the EstA AT of *P. stutzeri* A15. Apart from the β-barrel domain and the α-helical linker domain also the original esterase passenger domain was present in these modules, once with and once without the catalytic serine residue. The presence of the original passenger domain did not substantially resolve the problems encountered previously with the β-barrel domain based construct.

Also, in various cases smaller sized bands could be visualized. They could be indicative of translation intermediates or breakdown products. In *E. coli* it has been shown that surface exposed proteins can be proteolytically cleaved by the presence of proteases. Therefore, *E. coli* strains lacking the outer membrane protease (OmpT), like UT5600, are often preferred as host [12].

A first possible explanation for the differences between our results and reports describing successful surface display is the use of a different host organism. However, an often addressed issue for autodisplay in *E. coli*, the use of an for the host organism autologous AT, is nonetheless fulfilled [12]. Another reason could be the difference in viewpoint. Instead of focusing on the small fraction of passenger proteins that are proteinase K accessible and heat modifiable we concentrated on the major fraction of the proteins that were not secreted. The benchmark for successful surface display in our case was set by the data available for the surface display of the original EstA passenger domain using the β-barrel domain based construct. Others might have applied the term successful surface display in a less stringent way. Also, literature presumably has a bias towards the successful use of ATs for surface display. Despite the fact that the number of reports criticizing AT based surface display are limited, publications have reported the negative effect on membrane permeability [44] and on cell survival [45].

To end, it is important to mention that some reports are available which have addressed the nature of the passenger domain. The mechanism of translocation and secretion of passenger domains in the majority of these studies was only addressed by looking to the effects of introducing cysteine residues and the resulting disulfide bond formation. Some of the initial studies revealed the incompatibility of periplasmic disulfide bond formation with translocation of passenger domains [46-48]. These early reports were later contested by work describing the successful translocation of folded domains containing disulfide bonds [26,49,50]. The most recent studies on this topic however concluded that disulfide bond formation can only be tolerated if the cysteine residues are closely spaced [31,32]. Using a different approach it was suggested that the folding of a MalE passenger domain fused to the IgA AT interfered with its translocation across the outer membrane [51]. Also the introduction of the rigid and bulky calmodulin in the backbone of the Hbp AT has been shown to negatively affect secretion [32]. Finally it was proposed that secretion efficiency is dependent on the folding properties of the passenger domain itself [52]. Based on our results and the discussion above we suggest that more basic molecular knowledge of naturally occuring autotransporters is needed before such systems can be used for heterologous expression.

## Conclusion

We have shown that an expression module based on the β-domain of the *P. stutzeri* A15 EstA AT is not generally applicable for successful surface display of heterologous passenger domains in *P. stutzeri* A15. Essentially, the same holds true for expression modules based on the full-length EstA AT. In conclusion, the EstA AT cannot be seen as a broad applicable cell surface display system in *P. stutzeri* A15.

## Methods

### Bacterial strains and growth conditions

*E. coli* strain Top10 (Life Technologies) was used for cloning. For protein expression, *P. stutzeri* strain A15 [21,22] was used. Triparental conjugation of plasmids to *P. stutzeri* A15 was carried out with the helper plasmid pRK2073.

*P. stutzeri* A15 and *E. coli* strains were routinely cultured as described before [14]. If appropriate, antibiotics were added to the media in a final concentration of 10 μg ml^-1^ for tetracycline (Tc) and 50 μg ml^-1^ for spectinomycin (Sp).

### Construction of plasmids and general molecular biological techniques

All restriction enzymes (New England Biolabs), T4 DNA ligase (Life Technologies), Pfx DNA polymerase (Life Technologies), GenElute HP Plasmid Miniprep Kit (Sigma-Aldrich), GenElute PCR Clean Up Kit (Sigma-Aldrich), QIAEX II Gel Extraction Kit (Qiagen) and Puregene Genomic DNA Isolation Kit (Qiagen) were used according to the manufacturer’s instructions. Oligonucleotide primers were purchased from IDT DNA. All plasmids were sequenced by GATC Biotech to verify the nucleotide sequences.

All plasmids and primers used in this study are listed in Table S1 (Additional file 3) and Table S2 (Additional file 7), respectively. The plasmids constructed in this study have a pHERD26T [53] backbone. To construct the pEstA_β_ plasmid a number of intermediate constructs was created. The β-domain and part of the α-helical linker (from residue W328 to residue L636, residue numbering starting at the GTG start codon) of *estA* were PCR amplified with primers AI2981 and AI2982. This fragment was cloned in the HindIII/XbaI sites of pHERD26T. In the next step, the nucleotide sequence coding for the EstA signal peptide, 2 SfiI sites and the E-tag were introduced into the intermediary plasmid. Hereto a pUC19 fragment was PCR amplified using primers AI3505 and AI3507 and cloned into the vector in the XbaI/NcoI sites. The original pHERD26T SfiI site was furthermore removed by site directed mutagenesis with primers AI4569, AI4570, AI4571 and AI4572. Because this intermediary plasmid did not possess the entire α-helical linker, a new *estA* fragment was PCR amplified with primers AI4985 and AI4986 and cloned in the XbaI/SalI sites. This resulted in the pEstA_β_ plasmid (with the α-helical linker starting from residue A311). To construct pEstA_β_L, the 5’ UTR of pHERD26T-*estA* was PCR amplified with primers AI7328 and AI7329 and cloned into the XbaI/NcoI sites of pEstA_β_.

In the first step to create the pEstA and pEstA* plasmids an *estA* fragment without the signal peptide (from residue V20 to residue L636) was PCR amplified using primers AI2982 and AI2984. The PCR product was cloned in the HindIII/XbaI sites of pHERD26T. Hereafter the nucleotide sequence coding for the EstA signal peptide, 2 SfiI sites and the E-tag were added to the plasmid. This was done by PCR amplification of a pUC19 spacer fragment using the primers AI4184 and AI3507 and subsequent cloning in the XbaI/EcoRI sites. Also here the pHERD26T original SfiI site was afterwards removed by site directed mutagenesis. Because of a different cloning strategy the 5^′^ UTR of the resulting plasmid differs from the one present in pEstA_β_. To get similar expression levels for both plasmid series the pEstA_β_ 5^′^ UTR was also used for this plasmid series. In a first step a silent mutation removed the NcoI site present in the *estA* fragment of the plasmid using primers AI6276, AI6277, AI6278 and AI6280. In the resulting vector the XbaI/NcoI fragment was replaced by the XbaI/NcoI fragment of pEstA_β_ resulting in pEstA. In this plasmid the catalytic serine of EstA was substituted for an alanine by site directed mutagenesis using primers AI5083, AI5084, AI5085 and AI6280. This gave rise to pEstA*.

To create pEstA_β_-*aiiA*, pEstA_β_-*aiiB*, pEstA_β_-*attM*, pEstA_β_-*eGFP*, pEstA_β_-*mCherry*, pEstA_β_-*yEVenus* and pEstA_β_-*bla* the coding sequences of respectively *aiiA, aiiB, attM, eGFP, mCherry, yEVenus* and *bla* were PCR amplified with the respective primer pairs AI4567/AI4568, AI4958/AI4959, AI4956/AI4957, AI5247/AI5248, AI5247/AI5248, AI5245/AI5246 and AI7193/AI7194 on the respective DNA templates pMIR101, *Agrobacterium tumefaciens* C58 gDNA, *Agrobacterium tumefaciens* C58 gDNA, pET28a-*eGFP*, pET28a-*mCherry*, pKT103 and pUC19. The resulting fragments were cloned into the SfiI sites of pEstA_β_. In a similar way pEstA-*aiiA*, pEstA*-*aiiA*, pEstA-*aiiB*, pEstA*-*aiiB*, pEstA-*attM*, pEstA*-*attM*, pEstA-*eGFP*, pEstA*-*eGFP*, pEstA-*mCherry*, pEstA*-*mCherry*, pEstA-*yEVenus*, pEstA*-*yEVenus*, pEstA-*bla* and pEstA*-*bla* were constructed. pEstA_β_-*estAP*, pEstA-*estAP* and pEstA*-*estAP* were made by cloning the coding sequence of the esterase passenger domain of *estA* (from residue P27 to S325) into pEstA_β_, pEstA and pEstA* respectively.

### Protein expression and analysis

Expression of ORFs on the pHERD26T derivatives was under control of the p_BAD_ promoter. *P. stutzeri* A15 strains were grown at 30°C and 200 rpm in LB + Tc until OD_600_ of 0.8 was reached and 0.2% L-arabinose (final concentration) was added to start protein production. After induction, growth was continued at 23°C and 200 rpm for 3 hours.

For whole cell proteinase K digestion assays, 1.5 ml samples were resuspended in buffer (50 mM Tris pH 8, 7.5 mM CaCl_2_) to obtain an OD_600_ of 5. 100 μl samples were treated with a final concentration of 100 ng μl^-1^proteinase K (Fischer) or mock treated with distilled water and incubated for one hour at 37°C and 1400 rpm. To stop digestion 4 mM phenylmethanesulfonylfluoride was added. After centrifugation, the cell pellet was resuspended in 105 μl buffer (50 mM Tris pH 8, 4 mM phenylmethanesulfonylfluoride).

To test for beta-lactamase activity, induced cells were resuspended in 0.85% NaCl to obtain an OD_600_ of 1.5. A 0.5 mg ml^-1^ nitrocefin stock solution (2 mg nitrocefin, Calbiochem) was suspended in 1.9 ml 100 mM MOPS, 0.85% NaCl, pH 7 and 0.1 ml DMSO and was twenty-fold diluted in 100 mM MOPS, 0.85% NaCl, pH 7.90 μl was added to 10 μl of the bacterial suspension. The activity was measured by monitoring OD_486_ at 37°C. Esterase activity of whole cells or MFs was measured as described before [14]. MFs were prepared starting from 200 mg cell pellet as previously described [14].

For visualization, proteins were separated by SDS-PAGE (NuPAGE 10% Bis-Tris MOPS, Life Technologies) and detected with Western blotting. Prior to SDS-PAGE, samples were heated 10 min at 70°C unless otherwise stated. After electrophoresis, proteins were transferred onto polyvinylidene fluoride membranes (Life Technologies) by electroblotting (30V/60 min at 4°C). Membranes were incubated with the previously described rabbit anti-EstA serum (1:2000 dilution) [14], rabbit anti E-tag antibodies (1:2000 dilution, Abcam) or with rabbit anti-GroEL antibodies (1:2000 dilution, Sigma-Aldrich). Detection was done via alkaline-phosphatase conjugated goat anti-rabbit IgG secondary antibodies (Sigma-Aldrich) and NBT/BCIP (Nitro blue tetrazolium chloride/5-Bromo-4-chloro-3-indolylphosphate, Sigma).

## Abbreviations

AT: Autotransporter; EstAP: EstA passenger domain; EV: Empty Vector; MF: Membrane Fraction; PK: Proteinase K; UTR: Untranslated Region.

## Competing interests

The authors declare that they have no competing interests.

## Authors’ contributions

T.N., L.L, E.L. and S.B. designed and constructed the expression plasmids. T.N. performed enzymatic assays, membrane preparations and whole cell proteinase K assays. T.N., J.V. and S.S. designed the research project and wrote the manuscript. All authors read and approved the final manuscript.

## Supplementary Material

Additional file 1 **Figure S1. **Assessment of the fusion protein concentration in the membrane fractions of *P. stutzeri* A15 pHERD26T-*estA* or pEstAβL-*estAP.* Click here for file

Additional file 2 **Figure S2. **Heat modifiability analysis of proteins in the membrane fractions of *P. stutzeri *A15 pHERD26T-*estA*, pEstA_β_-*estAP* and pEstA_β_L-*estAP.* Click here for file

Additional file 3 **Table S1. **Plasmids used in this study. Click here for file

Additional file 4 **Figure S3. **Proteinase K accessibility of fusion proteins from *P. stutzeri* A15 pEstA_β_-*mCherry*/*eGFP*/*yEVenus.* Click here for file

Additional file 5 **Figures S4-S7. **Proteinase K accessibility of fusion proteins from *P. stutzeri* A15 pEstA/pEstA*-*aiiA*/*aiiB/attM/eGFP/mCherry/yEVenus/bla.* Click here for file

Additional file 6 **Figures S8-S10. **Heat modifiability analysis of proteins in the membrane fractions of *P. stutzeri *A15 pEstA*-*aiiA*/*aiiB*/*attM*/*bla*/*eGFP*/*mCherry*/*yEVenus *and pEstA-*eGFP*/*mCherry*/*yEVenus.* Click here for file

Additional file 7 **Table S2. **Primers used in this study. Click here for file
