# Supplementary material for: Probing the applicability of autotransporter based surface display with the EstA autotransporter of Pseudomonas stutzeri A15
Source: Microb Cell Fact. 2012 Dec 13;11:158. doi: 10.1186/1475-2859-11-158 (PMC3546941; doi:10.1186/1475-2859-11-158)
Supplement: Additional file 1 — Figure S1. Assessment of the fusion protein concentration in the membrane fractions of P. stutzeri A15 pHERD26T-estA or pEstAβL-estAP. [file 1475-2859-11-158-S1.pdf]

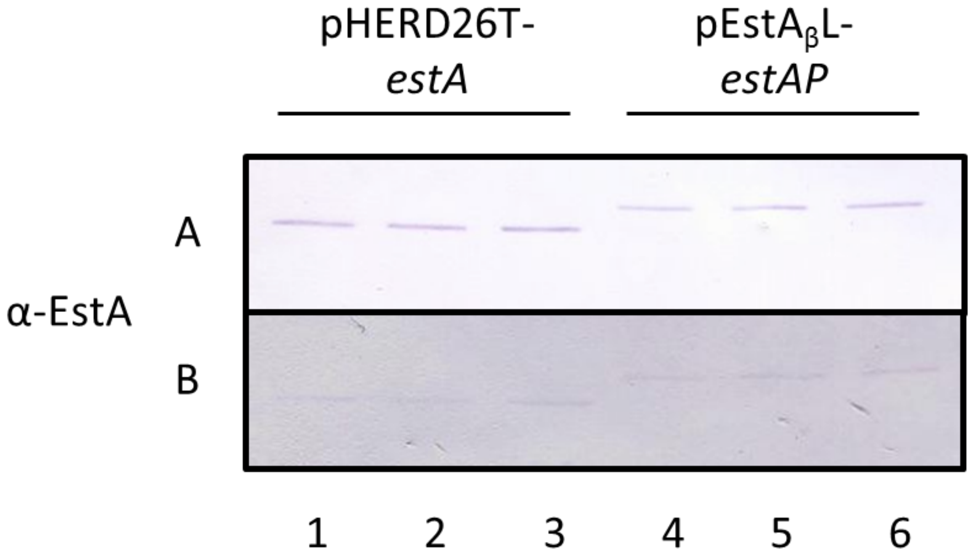

Figure S1: Assessment of the fusion protein concentration in the membrane fractions of *P. stutzeri* A15 pHERD26T-*estA* or pEstA$_{\beta}$L-*estAP*. Western blot with anti-EstA serum (α-EstA) of membrane fraction samples, used to measure the relative esterase activity of membrane fractions of *P. stutzeri* A15 pHERD26T-*estA* or pEstA$_{\beta}$L- *estAP* (Figure 1E), to show equal amounts of EstA/EstAP in the applied samples. Membrane fractions in B are 2.5 times diluted compared to A.
