# Supplementary material for: Probing the applicability of autotransporter based surface display with the EstA autotransporter of Pseudomonas stutzeri A15
Source: Microb Cell Fact. 2012 Dec 13;11:158. doi: 10.1186/1475-2859-11-158 (PMC3546941; doi:10.1186/1475-2859-11-158)
Supplement: Additional file 2 — Figure S2. Heat modifiability analysis of proteins in the membrane fractions of P. stutzeri A15 pHERD26T-estA, pEstAβ-estAP and pEstAβL-estAP. [file 1475-2859-11-158-S2.pdf]

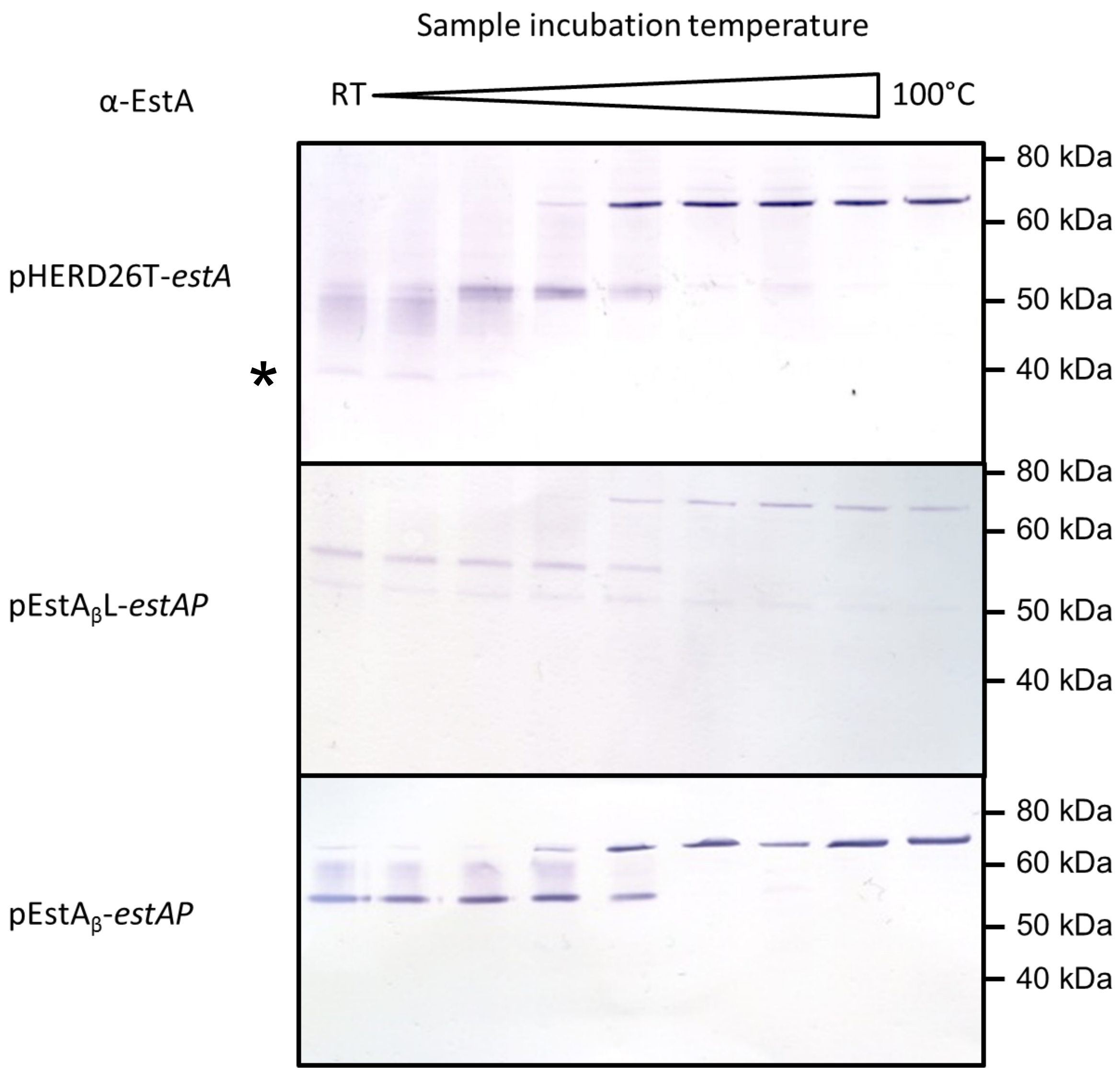

Figure S2: Heat modifiability analysis of proteins in the membrane fractions of *P. stutzeri* A15 pHERD26T-*estA*, pEstA$_{\beta}$-*estAP* and pEstA$_{\beta}$L-*estAP*. SDS-PAGE samples were incubated at different temperatures (room temperature [RT]-100°C) and analyzed with Western blot using anti-EstA serum (α-EstA). The passenger domain of proteins of pHERD26T-*estA* shows an additional heat modifiability, indicated with an *. Molecular weight markers are indicated at the side of the panels.
