# Supplementary material for: Probing the applicability of autotransporter based surface display with the EstA autotransporter of Pseudomonas stutzeri A15
Source: Microb Cell Fact. 2012 Dec 13;11:158. doi: 10.1186/1475-2859-11-158 (PMC3546941; doi:10.1186/1475-2859-11-158)
Supplement: Additional file 3 — Table S1. Plasmids used in this study. [file 1475-2859-11-158-S3.pdf]

Table S1 Plasmids used in this study

Plasmid	Relevant characteristics	Reference
pHERD26T	pBAD-promoter, Tc^R, pRO-1600 *ori*, pBR322 *ori*, *oriT*	[1]
pHERD26T-*estA*	pHERD26T containing *estA* in EcoRI and XbaI	[2]
$pEstA_{\beta}$	pHERD26T containing the *estA* signal peptide and β-domain (W328-L636), E-epitope tag and SfiI sites comprising a pUC19 fragment	This study
$pEstA_{\beta}$-*estAP*	$pEstA_{\beta}$ containing EstA passenger domain (EstAP; P27-S325) in SfiI sites	This study
$pEstA_{\beta}$-*bla*	$pEstA_{\beta}$ containing *bla* in SfiI sites	This study
$pEstA_{\beta}$-*aiiA*	$pEstA_{\beta}$ containing $aiiA_{soil}$ in SfiI sites	This study
$pEstA_{\beta}$-*aiiB*	$pEstA_{\beta}$ containing *aiiB* in SfiI sites	This study
$pEstA_{\beta}$-*attM*	$pEstA_{\beta}$ containing *attM* in SfiI sites	This study
$pEstA_{\beta}$-*mCherry*	$pEstA_{\beta}$ containing *mCherry* in SfiI sites	This study
$pEstA_{\beta}$-*eGFP*	$pEstA_{\beta}$ containing *eGFP* in SfiI sites	This study
$pEstA_{\beta}$-*yEVenus*	$pEstA_{\beta}$ containing *yEVenus* in SfiI sites	This study
pEstA	pHERD26T containing the *estA* signal peptide and full length *estA* AT (V20-L636), E-epitope tag and SfiI sites comprising a pUC19 fragment	This study
pEstA-*bla*	pEstA containing *bla* in SfiI sites	This study
pEstA-*aiiA*	pEstA containing $aiiA_{soil}$ in SfiI sites	This study
pEstA-*aiiB*	pEstA containing *aiiB* in SfiI sites	This study
pEstA-*attM*	pEstA containing *attM* in SfiI sites	This study
pEstA-*mCherry*	pEstA containing *mCherry* in SfiI sites	This study
pEstA-*eGFP*	pEstA containing *eGFP* in SfiI sites	This study
pEstA-*yEVenus*	pEstA containing *yEVenus* in SfiI sites	This study
pEstA*	pEstA carrying S-A mutation in the catalytic triad of the EstA passenger domain	This study
pEstA*-*bla*	pEstA* containing *bla* in SfiI sites	This study
pEstA*-*aiiA*	pEstA* containing $aiiA_{soil}$ in SfiI sites	This study
pEstA*-*aiiB*	pEstA* containing *aiiB* in SfiI sites	This study
pEstA*-*attM*	pEstA* containing *attM* in SfiI sites	This study
pEstA*-*mCherry*	pEstA* containing *mCherry* in SfiI sites	This study
pEstA*-*eGFP*	pEstA* containing *eGFP* in SfiI sites	This study
pEstA*-*yEVenus*	pEstA* containing *yEVenus* in SfiI sites	This study
pRK2073	Sp^R, Tra+, Mob+, ColEI replicon	[3]
pUC19	Ap^R, ColEI replicon	[4]
pMIR101	$aiiA_{soil}$ containing vector, Km^R	[5]
pKT103	*yEVenus* containing vector, Km^R	[6]
pET28a-*mCherry*	*mCherry* containing vector, Km^R	[7]
pET28a-*eGFP*	*eGFP* containing vector, Km^R	[8]

[1] Qiu DR, Damron FH, Mima T, Schweizer HP, Yu HD: **P-BAD-based shuttle vectors for functional analysis of toxic and highly regulated genes in Pseudomonas and Burkholderia spp. and other bacteria.** *Appl Environ Microbiol* 2008, **74**(23):7422–7426.
[2] Nicolay T, Devleeschouwer K, Vanderleyden J, Spaepen S: **Characterization of Esterase A, a *Pseudomonas stutzeri* A15 autotransporter.** *Appl Environ Microbiol* 2012, **78**(8):2533–2542.
[3] Figurski DH, Helinski DR: **Replication of an origin-containing derivative of plasmid RK2 dependent on a plasmid function provided in trans.** *P Natl Acad Sci USA* 1979, **76**(4):1648–1652.
[4] Yanisch-Perron C, Vieira J, Messing J: **Improved M13 phage cloning vectors and host strains: Nucleotide sequences of the M13mp18 and pUC19 vectors.** *Gene* 1985, **33**(1):103–119.
[5] Carlier A, Uroz S, Smadja B, Fray R, Latour X, Dessaux Y, Faure D: **The Ti plasmid of *Agrobacterium tumefaciens* harbors an *attM*-paralogous gene, *aiiB*, also encoding N-acyl homoserine lactonase activity.** *Appl Environ Microbiol* 2003, **69**(8):4989–4993.
[6] Sheff MA, Thorn KS: **Optimized cassettes for fluorescent protein tagging in *Saccharomyces cerevisiae*.** *Yeast* 2004, **21**(8):661–670.
[7] De Meulenaere E, De Wergifosse M, Botek E, Spaepen S, Champagne B, Vanderleyden J, Clays K: **Nonlinear optical properties of mStrawberry and mCherry for second harmonic imaging.** *Journal of Nonlinear Optical Physics & Materials* 2010, **19**(1):1–13.
[8] De Meulenaere E, Asselberghs I, de Wergifosse M, Botek E, Spaepen S, Champagne B, Vanderleyden J, Clays K: **Second-order nonlinear optical properties of fluorescent proteins for second-harmonic imaging.** *J Mater Chem* 2009, **19**(40):7514–7519.
