# Supplementary material for: Probing the applicability of autotransporter based surface display with the EstA autotransporter of Pseudomonas stutzeri A15
Source: Microb Cell Fact. 2012 Dec 13;11:158. doi: 10.1186/1475-2859-11-158 (PMC3546941; doi:10.1186/1475-2859-11-158)
Supplement: Additional file 4 — Figure S3. Proteinase K accessibility of fusion proteins from P. stutzeri A15 pEstAβ-mCherry/eGFP/yEVenus. [file 1475-2859-11-158-S4.pdf]

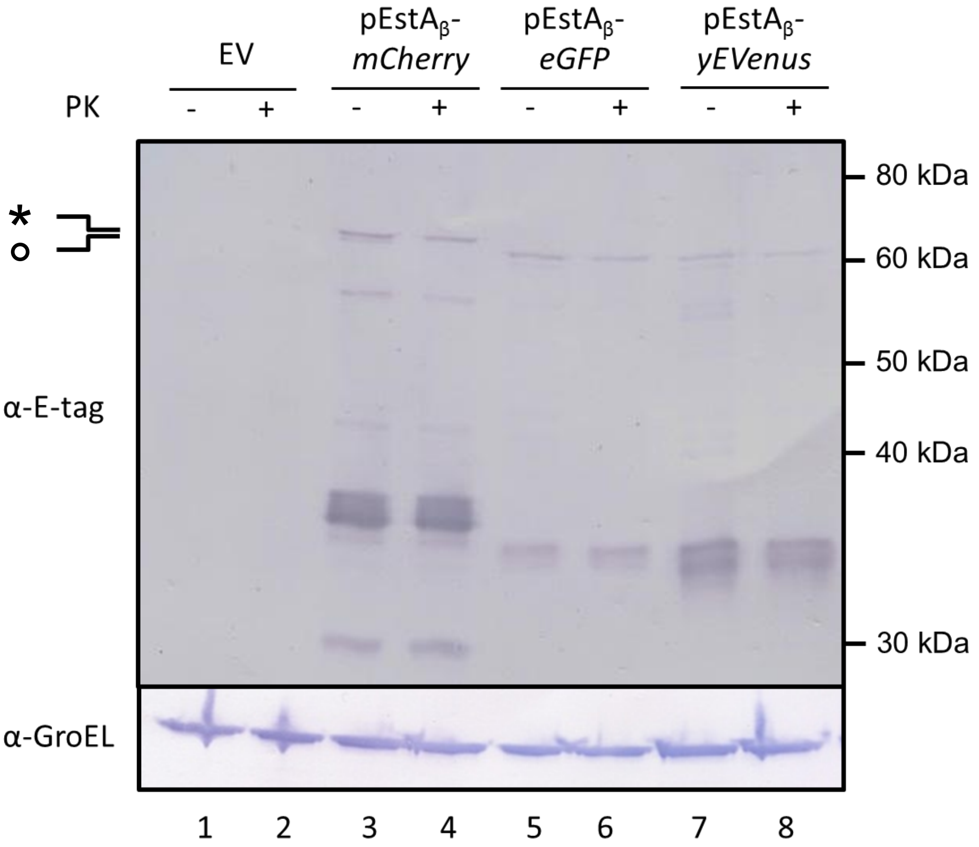

Figure S3: Proteinase K accessibility of fusion proteins from *P. stutzeri* A15 pEstA_β-*mCherry/eGFP/yEVenus.* Induced cells of *P. stutzeri* A15 pEstA_β-*mCherry/eGFP/yEVenus* or empty vector (EV) were treated with proteinase K (PK +) or mock-treated (PK -). Samples were analyzed with Western blot using anti-E-tag antibodies (α-E-tag) or anti-GroEL antibodies (α-GroEL). Molecular weight markers are indicated at the side of the panels.
