# Supplementary material for: Probing the applicability of autotransporter based surface display with the EstA autotransporter of Pseudomonas stutzeri A15
Source: Microb Cell Fact. 2012 Dec 13;11:158. doi: 10.1186/1475-2859-11-158 (PMC3546941; doi:10.1186/1475-2859-11-158)
Supplement: Additional file 5 — Figures S4-S7. Proteinase K accessibility of fusion proteins from P. stutzeri A15 pEstA/pEstA*-aiiA/aiiB/attM/eGFP/mCherry/yEVenus/bla. [file 1475-2859-11-158-S5.pdf]

S4

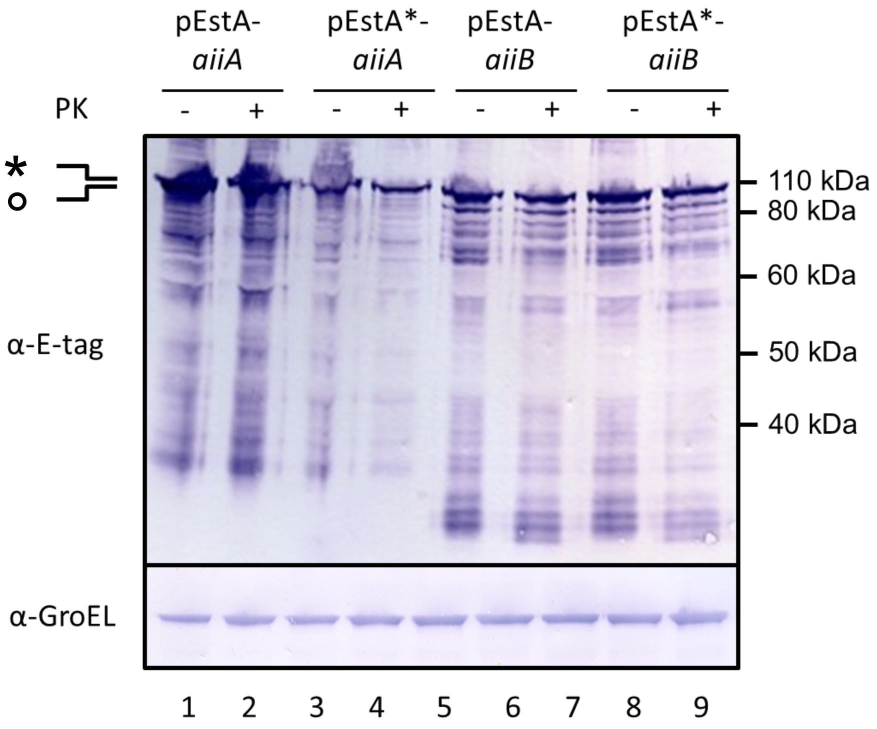


S5

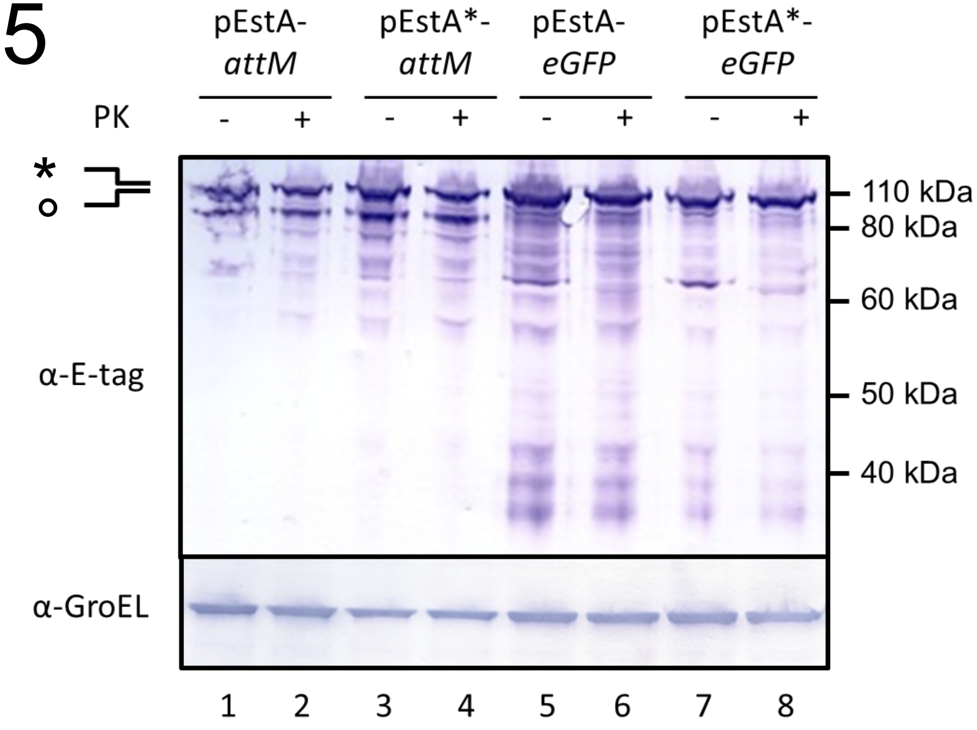


S6

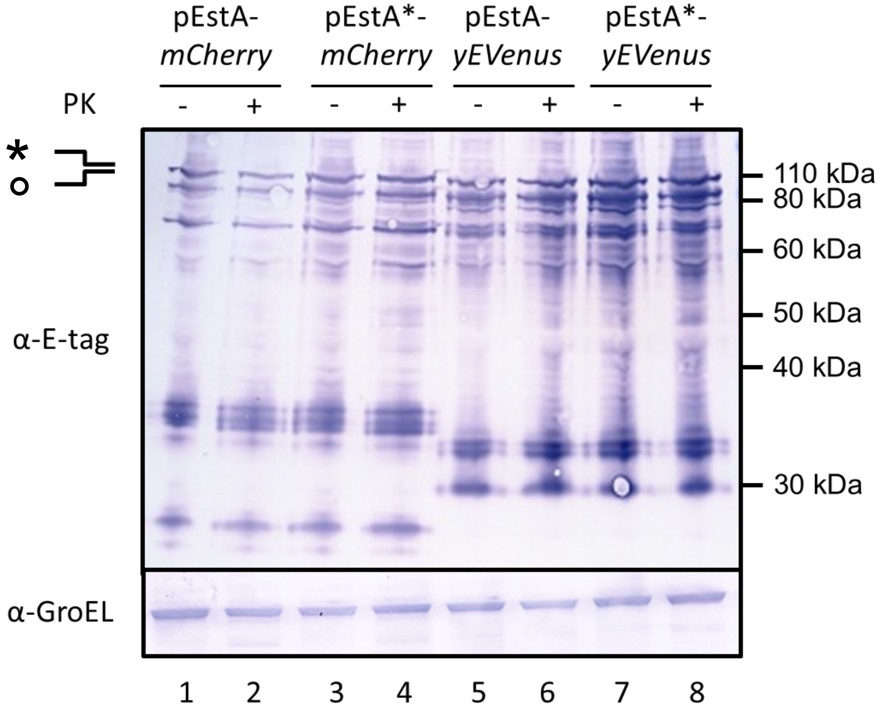


S7

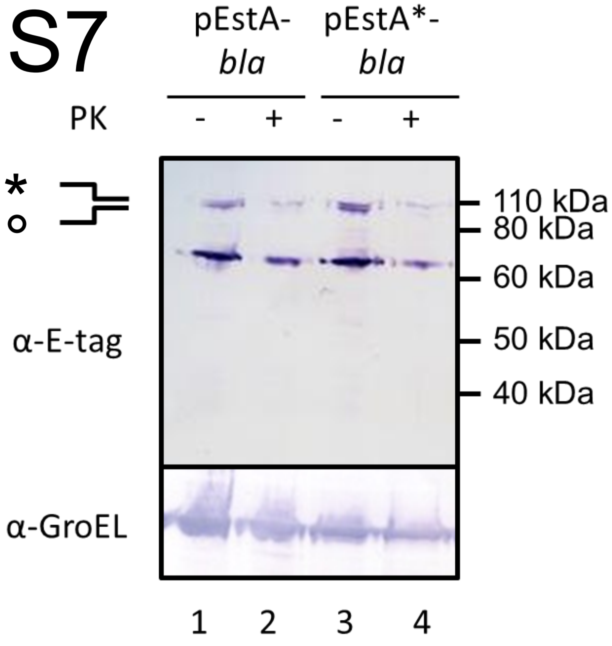


**Figures S4-S7: Proteinase K accessibility of fusion proteins from *P. stutzeri* A15 pEstA/pEstA*-*aiiA/aiiB/attM/eGFP/mCherry/yEVenus/bla.*** Induced cells of *P. stutzeri* A15 pEstA/pEstA*-*aiiA*/*aiiB* (S4), pEstA/pEstA*-*attM*/*eGFP* (S5), pEstA/pEstA*- *mCherry*/*yEVenus* (S6) and pEstA/pEstA*-*bla* (S7) were treated with proteinase K (PK +) or mock-treated (PK -). Samples were analyzed with Western blot using anti-E-tag antibodies (α-E-tag). The band with the higher apparent molecular weight, containing the signal peptide, is indicated with a '*'. The band with the lower apparent molecular weight, without the signal peptide is indicated with an 'o'. Molecular weight markers are indicated at the side of the panels.
