# Supplementary material for: Probing the applicability of autotransporter based surface display with the EstA autotransporter of Pseudomonas stutzeri A15
Source: Microb Cell Fact. 2012 Dec 13;11:158. doi: 10.1186/1475-2859-11-158 (PMC3546941; doi:10.1186/1475-2859-11-158)
Supplement: Additional file 6 — Figures S8-S10. Heat modifiability analysis of proteins in the membrane fractions of P. stutzeri A15 pEstA*-aiiA/aiiB/attM/bla/eGFP/mCherry/yEVenus and pEstA-eGFP/mCherry/yEVenus. [file 1475-2859-11-158-S6.pdf]

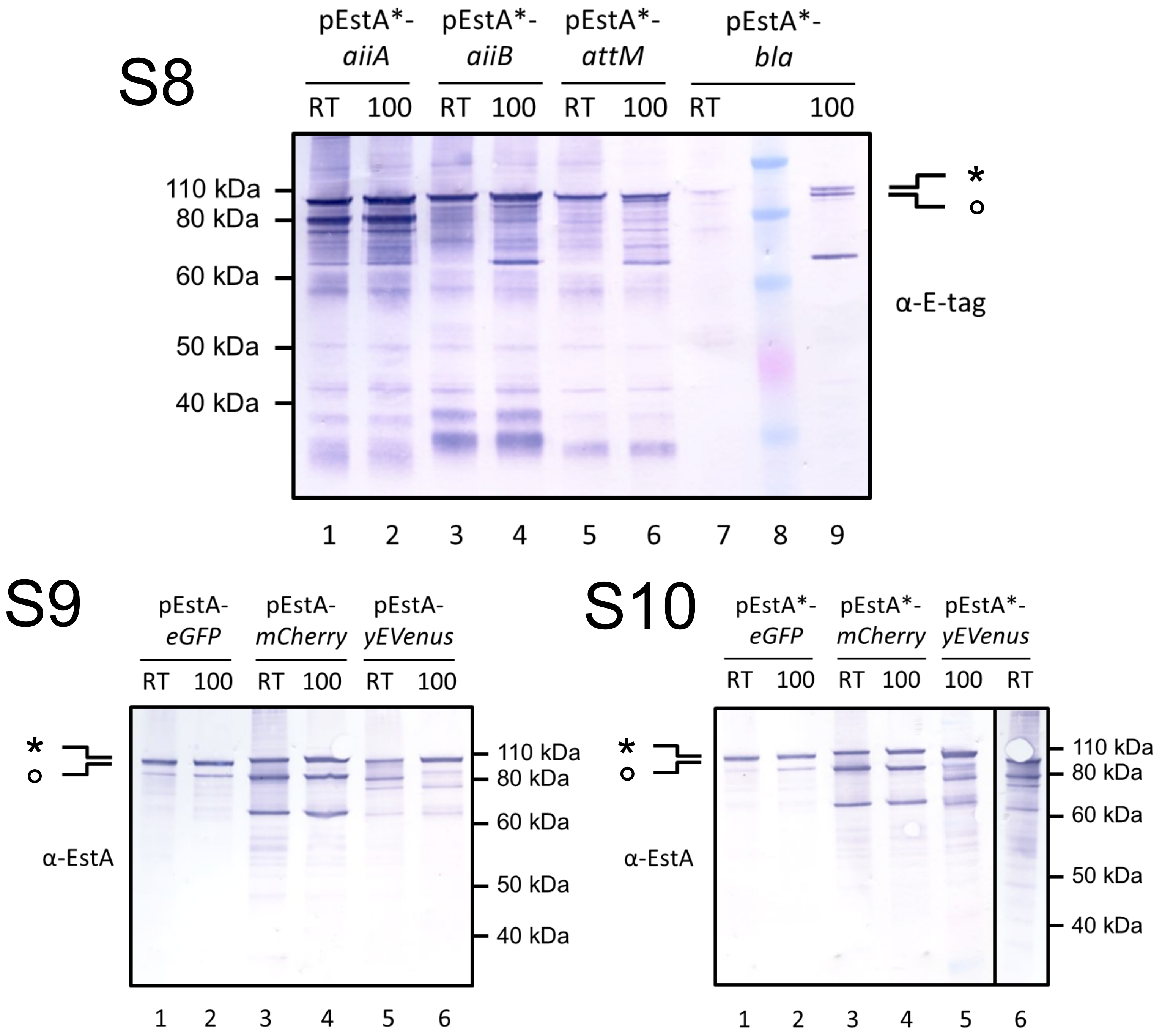

Figures S8-S10: Heat modifiability analysis of proteins in the membrane fractions of *P. stutzeri* A15 pEstA*-*aiiA/aiiB/attM/bla/eGFP/mCherry/yEVenus* and pEstA-*eGFP/mCherry/yEVenus*. Fusion proteins in the membrane fractions of induced cells of *P. stutzeri* A15 pEstA*-*aiiA/aiiB/attM/bla* (S8), pEstA-*eGFP/mCherry/yEVenus* (S9) and pEstA*-*eGFP/mCherry/yEVenus* (S10) were tested for heat modifiability. SDS-PAGE samples were incubated at room temperature (RT) or 100°C and analyzed with Western blot using anti E-tag antibodies (α-E-tag) or anti-EstA serum (α-EstA). The band with the higher apparent molecular weight, containing the signal peptide, is indicated with a '∗'. The band with the lower apparent molecular weight, without the signal peptide is indicated with an 'o'. Molecular weight markers are indicated at the side of the panels.
