# Supplementary material for: Probing the applicability of autotransporter based surface display with the EstA autotransporter of Pseudomonas stutzeri A15
Source: Microb Cell Fact. 2012 Dec 13;11:158. doi: 10.1186/1475-2859-11-158 (PMC3546941; doi:10.1186/1475-2859-11-158)
Supplement: Additional file 7 — Table S2. Primers used in this study. [file 1475-2859-11-158-S7.pdf]

Table S2: Primers used in this study

Primer	Sequence (5' - 3')
AI2981	ACTGTCTAGATGGGAAGTCTCGCTGCTG
AI2982	ACTGAAGCTTCTCCCTCTACAGATCCAAGGC
AI2984	ACTGTCTAGAGTCGCTACTGCCGATAGC
AI3505	ACTGCCATGGCACAAAGCGCTCTCAAGCCGCTTGCGGCAGCCGTTCTGCTCACCTGCGTGTCCAGC GTCGCTACTGCCGATCGGCCCAGCCGGCCATTCCACACAACATACGAGC
AI3507	ACTGTCTAGAGCGCGGCTCCAGCGGGTCCGGGTACGGCACCGGGGCGCCGGCCTCGGGGGCCGT TCTTTCCTGCGTTATCCC
AI4184	ACTGGAATTCATAAATGCAAAGCGCTCTCAAGCCGCTTGCGGCAGCCGTTCTGCTCACCTGCGTGT CCAGCGTCGCTACTGCCGATCGGCCCAGCCGGCCATTCCACACAACATACGAGC
AI4567	ACTGGGCCCAGCCGGCCTACAGTAAAGAAGCTTTATTTCGTACCAGC
AI4568	ACTGGGCCTCGGGGGCCCCTATATATTCAGGGAACACTTTACAACTCTTTTCC
AI4569	GCCTTGGATCTGTAGAGGGAG
AI4570	TTCAGGGGCACAAATGCG
AI4571	CCGTGGCCACCACAGCCCGGCCTGC
AI4572	GCAGGCCGGGCTGTGGTGGCCACGG
AI4956	ACTGGGCCTCGGGGGCCCCCGCGTAAAATTCGGGAGCC
AI4957	ACTGGGCCCAGCCGGCCTCTTCAGTCGGGTACGCTG
AI4958	ACTGGGCCTCGGGGGCCCCTTCATAGAATCCGTCCGTGGATTTG
AI4959	ACTGGGCCCAGCCGGCCTGGAAATAAGCTGTTCGTTCTCGACC
AI4985	ACTGTCTAGAGCGGTTCACCAGATCAGC
AI4986	ACTGGTCGACGTCCACTTTCTGG
AI5083	CGCATCGCTCAGCGCGTCGCCGAATACGATG
AI5084	CATCGTATTCGGCGACGCGCTGAGCGATGCG
AI5085	CGCTCAGGAAGTTGAACGTCG
AI5245	ACTGGGCCCAGCCGGCCTTCTAAAGGTGAAGAATTATTCACTGGTG
AI5246	ACTGGGCCTCGGGGGCCCCTTTGTACAATTCATCCATACCATGGG
AI5247	ACTGGGCCCAGCCGGCCTGTGAGCAAGGGCGAGGAG
AI5248	ACTGGGCCTCGGGGGCCCCCTTGTACAGCTCGTCCATGCC
AI6276	GCGGCTTGGGCCATCGTCACCGGGTTGGTG
AI6277	CACCAACCCGGTGACGATGGCCCAAGCCGC
AI6278	GTCGCTCAGGAAGTTGAACG
AI6280	CGCTGGATTTGATTCGGAATTAGC
AI7193	ACTGGGCCCAGCCGGCCTATGAGTATTCAACATTTCCGTGTCGC
AI7194	ACTGGGCCTCGGGGGCCCCCCAATGCTTAATCAGTGAGGCAC
AI7328	GATCCCATGGGATCTGATAAGAATTCGTAAGAGATACCCCCGTGCAAAGCGCTCTCAAGCCG
AI7329	GATCTCTAGAGCGCGGCTCC
